# The definition of sexual selection

**DOI:** 10.1093/beheco/arab055

**Published:** 2021-08-07

**Authors:** David M Shuker, Charlotta Kvarnemo

**Affiliations:** 1 School of Biology, Harold Mitchell Building, University of St. Andrews, St. Andrews, UK; 2 Department of Biological and Environmental Sciences, University of Gothenburg, Gothenburg, SE, Sweden

**Keywords:** natural selection, sexual selection

## Abstract

Sexual selection is a key component of evolutionary biology. However, from the very formulation of sexual selection by Darwin, the nature and extent of sexual selection have been controversial. Recently, such controversy has led back to the fundamental question of just what sexual selection is. This has included how we incorporate female-female reproductive competition into sexual or natural selection. In this review, we do four things. First, we examine what we want a definition to do. Second, we define sexual selection: *sexual selection is any selection that arises from fitness differences associated with nonrandom success in the competition for access to gametes for fertilization.* An important outcome of this is that as mates often also offer access to resources, when those resources are the targets of the competition, rather than their gametes, the process should be considered natural rather than sexual selection. We believe this definition encapsulates both much of Darwin’s original thinking about sexual selection, and much of how contemporary biologists use the concept of sexual selection. Third, we address alternative definitions, focusing in some detail on the role of female reproductive competition. Fourth, we challenge our definition with a number of scenarios, for instance where natural and sexual selection may align (as in some forms of endurance rivalry), or where differential allocation means teasing apart how fecundity and access to gametes influence fitness. In conclusion, we emphasize that whilst the ecological realities of sexual selection are likely to be complex, the definition of sexual selection is rather simple.

## Teaser

Sexual selection is a crucial component of evolutionary biology yet debate over what forms of selection do or don’t comprise sexual selection have re-surfaced over the last couple of decades. Here we provide and justify a definition of sexual selection, that is focused on competition for access to gametes, and challenge that definition with ecologically complex scenarios. We hope this will allow the field to move forward, building on a firm conceptual basis.

## INTRODUCTION

Sexual selection has a contentious history. Originally, sexual selection was proposed by Darwin (1859, 1871) to explain the evolution of traits that do not appear to increase longevity or fecundity, and so would not be favored by natural selection. Whilst the rest of the Darwinian view of evolution has been widely accepted, sexual selection has remained a focus of debate and argument for more than a century and a half (Cronin 1991; Andersson 1994; Kokko et al. 2006; Alonzo and Servedio 2019). First, the very notion of sexual selection itself proved less acceptable to early evolutionary biologists than natural selection, no doubt in part due to social attitudes at the time. For instance, Darwin removed the very term “sexual selection” from the title of *The Descent of Man* at the behest of his publisher John Murray, leaving instead the rather mangled “selection in relation to sex” (Dawson 2007), which has perhaps led to some misinterpretations of Darwin’s ideas (see below).

Second, Darwin was clearly aware of the potentially scandalous nature of sexual selection, and especially the idea of mate preferences. For example, he left the discussion of some human male mate preferences for certain female morphological attributes to a footnote written in Latin (Darwin 1871, p. 345; Dawson 2007). Mate choice in particular failed to gain widespread acceptance with Darwin’s contemporaries and those who came after. This may have been because Darwin emphasized female mate choice, and the idea of female animals expressing an “aesthetic taste” may have been hard to swallow, although there were notable exceptions (for example Peckham and Peckham 1889, 1890; see also Cronin 1991; Hamlin 2014). Certainly, the idea of male–male competition was more widely accepted, not least because male combat for mates was self-evident in animals such as rutting ungulates or horned beetles (Emlen 2008, 2014; for various perspectives on the history of sexual selection see for instance Cronin 1991, Andersson 1994, and Milam 2010).

Third, the resurgence of interest in sexual selection in the 1970s and 1980s (for example Campbell 1972; Blum and Blum 1979; Bateson 1983; Thornhill and Alcock 1983; Smith 1984; Bradbury and Andersson 1987; reviewed by Andersson 1994) also brought forth controversy. This resurgence included the rehabilitation of mate choice as a potent evolutionary mechanism, on the back of ground-breaking theoretical and empirical papers (Lande 1981; Andersson 1982; Kirkpatrick 1982). However, why those mate preferences evolved has proved challenging for theorists to understand and for empiricists to test. Mate choice associated with direct benefits (such as access to parental care, resources, or territories) has always been relatively straightforward to comprehend (Andersson 1994). Mate choice when males only provide sperm to females, and thus genes to offspring, remains contentious though (for recent discussions see Ingleby et al. 2010; Prum 2010; Hunt and Hosken 2014; Rosenthal 2017; Achorn and Rosenthal 2020).

Fourth, the conceptual basis of sexual selection was more recently challenged in a highly controversial paper (Roughgarden et al. 2006 and resulting commentaries). Whilst much of that paper has had little bearing on what sexual selection researchers do and think, perhaps the most relevant fall-out was attempts to redefine or reinterpret what sexual selection actually is. This has included reconsidering the role of females in sexual selection (for example LeBas 2006; Clutton-Brock 2007, 2009, 2017; Rosvall 2011; Stockley and Bro-Jørgensen 2011; Tobias et al. 2012; Hare and Simmons 2019) and also the consideration of new definitions of sexual selection (for example Carranza 2009; see discussion in Shuker 2010, 2014; Alonzo and Servedio 2019).

Here we will explore definitions of sexual selection, provide what we think is the most suitable definition, and attempt to bring conceptual clarity to a concept about which there has perhaps been some intellectual complacency. We will begin by considering what a definition of sexual selection should and should not be able to achieve, then we will provide a definition, which will be familiar to many readers, before discussing alternative definitions and finally cases that push our definition to the limit. We will need to discuss how natural and sexual selection interact, and to do so we will follow Endler (1986) by considering sexual selection to be a component of “broad-sense” natural selection. “Narrow-sense” natural selection will be used to describe other components of fitness, such as longevity, fecundity, and parental investment (see below for a fuller discussion of this terminology).

## WHAT TO EXPECT FROM A DEFINITION OF SEXUAL SELECTION

A definition of sexual selection should fulfill a number of criteria. First, it needs to be sex neutral (that is, not dependent on a given sex or sexual function). This is because both males and females may present traits that are under sexual selection, even in the same species. Moreover, in species in which there are no sexes, that is isogamous species such as *Saccharomyces* yeasts, individuals within a mating type may compete for mates in a way that recalls sexual selection in anisogamous species (for example Rogers et al. 2009; Reding et al. 2013). In other words, a definition of sexual selection should require neither sexes nor specific sex roles (Shuker 2010, 2014).

Second, a definition of sexual selection should identify – in general terms – what individuals are competing for. Competition is important here. DNA sequences are competing for representation in the next generation, competition that can occur even if a population is expanding rapidly in size. An attempt to divorce sexual selection from the notion of competition was part of the critique of sexual selection put forward by Roughgarden et al. (2006). Those authors wanted to view interactions between individuals in terms of reproduction as cooperative, with males and females working together as a “team.” However, cooperation – like selfishness – is just another strategy by which organisms may increase their genetic representation in the next generation, *at the expense of others*. After all, cooperation is in the eye of the beholder – your cooperation may be my cronyism and corruption. Putting forward cooperation in opposition to competition is therefore misguided and misinterprets levels of explanation. Darwin himself made it clear that selection is all about doing better than rivals, whether that be through teamwork or aggression.

On the other hand, there are some things that we should not expect a definition of sexual selection to be able to sort out for us. To begin with, sexual selection should be agnostic about (that is, unconcerned with) other components of fitness. In other words, it should not be defined in terms of other forms of selection. If it is, then sexual selection risks becoming contingent on other forms of selection that may or may not be present, which does not seem satisfactory to us. This might seem a reasonable proposition, but Darwin of course mentioned sexual selection in terms of traits that natural selection did not appear to favor (Darwin 1871, for example pp 278-279). This opposition to natural selection is still often emphasized, for instance when natural selection halts the exaggeration of sexually selected ornaments in models of mate choice (Lande 1981; Andersson 1994). However, when he introduced sexual selection in the first edition of *The Origin*, Darwin noted from the outset that males with the greatest “general vigour” (that is, most favored by natural selection) may also be the most successful at winning mates (Darwin 1859). Here, natural and sexual selection both favor “general vigour” and are not in opposition. What this means is that empirically we may not necessarily be able to discriminate between fitness associated with sexual selection, and fitness associated with other components of selection, if those fitness components *partially or completely align* (Darwin 1871, p. 257; Andersson 1994). However, it is an empirical problem that we should expect to face, given the countless ways in which components of fitness may instantiate themselves across the tangled bank of ecologies organisms inhabit in the wild.

Many mechanisms can result in sexual selection (Table 1). However, a definition of sexual selection should not be tied to one or more mechanisms of sexual selection, such as mate choice or male–male competition. This is important, as much of the Roughgarden et al (2006) critique focused on sexual selection through female mate choice, and indeed other recent reviews have tended to equate the two (for example Kuijper et al. 2012; a similar point was made by McCullough et al. 2016). Arguments over mate choice for instance – however prominent historically and contemporaneously – should not distract us from a more fundamental definition of sexual selection. Indeed, clarity over the definition of sexual selection may help the field from becoming preoccupied with one or other form of sexual selection.

**Table 1:** Mechanisms of Competition Over Mates and Gametes, Based on and Developed from Table 1.1.1 in Andersson (1994)

Mechanisms	Favoured traits in competing sex
Scramble competition	sensory and locomotory organs to quickly locate mates (for example hearing, olfaction)
Endurance rivalry	ability to endure prolonged reproductive activity (for example condition, lifespan)
Contest competition	ability to outcompete rivals before mating through direct combat (for example body size, weapons), or ability to avoid such competition through alternative reproductive tactics
Mate choice	competition to be chosen through behavioral or morphological traits that the opposite sex finds attractive (for example ornaments, indicators of ‘good’ or compatible genes), or resources that the other sex needs (for example territory, nuptial gift, parental care), or ability to circumvent mate choice (for example forced copulations)
Gamete competition after mating	ability to outcompete rivals through gamete competition after mating (for example large numbers of sperm, large size of eggs), or ability to avoid that gamete competition (for example mate guarding, mating plugs)
Cryptic mate choice	competition to be chosen after mating through traits that the opposite sex prefers (for example ‘good’ or compatible genes)

This is not to say that we would not like a body of theory that makes specific predictions about how sexual selection arises, or indeed that predicts specific traits or trait values favored by sexual selection. Fortunately, we do have that body of theory though, both in terms of general frameworks like mating systems theory (for example Emlen and Oring 1977; Davies 1991; Reynolds 1996; Shuster and Wade 2003; Shuker 2010; Kokko et al. 2014) and in terms of specific optimality models predicting sexually selected traits, such as copulation duration in dung flies (for example Parker 1970a; Parker and Simmons 1994) or time of emergence (protandry) in butterflies (for example Wiklund and Fagerström 1977; Bulmer 1983; Iwasa et al. 1983; Morbey and Ydenberg 2001; Morbey et al. 2012). Here, the analogy with the rest of natural selection theory is clear. For instance, we have a wonderful body of evolutionary theory used to predict and explain many of the traits we see, such as sex allocation theory (West 2009), but we do not include those specifics in our definition of natural selection.

Finally, we suggest that a definition should be agnostic about the number of mates individuals have, and indeed the nature and extent of variation in the number of mates or resulting fertilizations that individuals within a sex exhibit. A straightforward reason for this is that sexual selection can still occur in fully monogamous species (Kirkpatrick et al. 1990; Andersson 1994; Dougherty et al. 2016; Kvarnemo 2018), for example, if the adult sex ratio is biased (generating strong mating competition within the sex in excess; for example Price 1984; Kvarnemo et al. 2007; Fromhage and Jennions 2016), or through assortative pair-formation, often driven by mutual mate choice (for example Parker 1983; Johnstone et al. 1996; Jones and Ratterman 2009). We will consider variation in the number of mates in more detail below when we address definitions of sexual selection based on the variance in mating success.

## A DEFINITION OF SEXUAL SELECTION

After what we see as a continuity from the words and works of Darwin, as captured by Andersson (1994) and Shuker (2010, 2014), we define sexual selection as follows:

*Sexual selection is any selection that arises from fitness differences associated with nonrandom success in the competition for access to gametes for fertilization.*

Fertilization is important, hence, by “access to gametes” (as used as shorthand below) we emphasize that the route to fitness through competition for access to gametes requires fertilization as the outcome. For completeness, we assume that gametes are viable and able to take part in fertilization; this primary function of male and female gametes will be under natural selection. As such, a trait is under sexual selection when variation in that trait is nonrandomly associated with variation in access to gametes, when those gametes form a limiting resource. Evolution by sexual selection follows when there is a heritable component to the trait under sexual selection.

Indeed, in more genetical terms, the extent to which a given allele is under sexual selection is the extent to which there is a *marginal change in allele frequency* associated with nonrandom access to gametes for fertilization. Sexual selection is therefore one way of partitioning allele frequency change – that is into natural and sexual selection components of fitness – in an analogous way to partitioning allele frequency change into direct and indirect components of fitness in an inclusive fitness framework (for example Gardner et al. 2011). Henceforth we will assume additive genetic variation in relevant traits, to make the discussion more concise.

From the outset, we do not wish to suggest that our definition is new; indeed, our definition is very close to definitions of sexual selection given before (see, for instance, Kokko et al. 2006). We hope to make this abundantly clear when we review other definitions below. However, we consider that there are problems with alternative definitions of sexual selection and that the underlying logic of sexual selection is not always fully appreciated.

As such, several important points emerge from this definition. First, competition is for access to *gametes*. We recognize that access to *mates* is a typical first step towards obtaining access to gametes (in particular in internal fertilizers), and that we indeed expect two broad classes of sexual selection fitness components: those associated with precopulatory mating success (for example Bateson 1983; Hardy and Briffa 2013) and those associated with post-copulatory fertilization success (for example Parker 1970b; Smith 1984; Eberhard 1996; Birkhead and Møller 1998; Simmons 2001). As discussed by Birkhead (2010), it was this second component that was elusive to Darwin. However, the traditional focus on access to mates as the keystone to sexual selection is perhaps because humans are not broadcast spawners. We will return to the case when competition for mates may *not* yield sexual selection below.

Importantly, gametes as a resource can *vary in two ways*, and thus be *limiting in two ways*. They can vary in quantity, and they can vary in quality. Competition can therefore arise for access to gametes in terms of getting access to the most gametes, but also in terms of access to gametes of the highest quality. We will revisit the importance of competition in terms of quality below (as this is one of the areas where things can get hairy).

Second, we are agnostic as to the sexual identity of the competitors (it could be males, females, hermaphrodites, or individuals of mating type A or mating type B). This means that we are also agnostic as to whether or not sexual selection happens in both sexes/mating types at the same time; all options are possible. However, implicit in our definition is that individuals of the same sex, same mating type, or tissues with the same sexual function (for example male function in hermaphrodites), compete for the *gametes of the opposite sex* or complementary mating type. In other words, sexual selection arises from competition *within* a sex or mating type.

Whilst this again might seem straightforward, confusion can arise when sexual selection is influenced by interactions between the sexes. Such interactions certainly influence fertilization success, through mate choice (when one sex competes to be chosen by the other sex). But care is needed in noting which sex is competing for whose gametes.

The definition of mate choice has itself been recently reassessed (Edward 2014). We consider mate choice to arise from the nonrandom patterns of mating, and subsequent fertilization success, in the *chosen* sex due to one or more phenotypic traits in the *choosy* sex. These phenotypic traits may be behavioral (and approach what we might think of as a more active “choice”) or be morphological or physiological; what is important is that the chooser biases mating and/or fertilization as a result. Crucially, whilst there must be nonrandom success with respect to some aspect of the phenotype of the chosen sex for choice to occur, there need not be any variation amongst choosers (that is they may all choose the same individual or individuals). Mate choice thus mediates a form of within-sex competition for access to gametes, but it does not replace it.

Our definition is sex neutral, but it does identify what is being competed for (gametes for fertilization), albeit in general terms. Whilst we highlight competition for mates and gametes as two major classes of sexual selection fitness components, we have not identified specific mechanisms by which sexually selected fitness may be obtained. This also means that we have not specified whether or not sexual selection aligns with other components of fitness. As such, our definition is not contingent on the action of any other forms of selection. That said, we expect that sexual selection will comprise only some of the variation in fitness in a given population at a given time.

## THE RELATIONSHIP BETWEEN NATURAL SELECTION AND SEXUAL SELECTION

By being agnostic to other components of fitness, where does our definition place sexual selection with regards to natural selection? The relationship between natural and sexual selection has been long debated, from Darwin onwards (for example Darwin 1871; Fisher 1930; Arnold 1983; Andersson 1994; Klug et al. 2010; Alonzo and Servedio 2019). As mentioned above, a particularly useful conceptualization was provided by Endler (1986). He suggested that *broad-sense* natural selection should be taken to comprise all possible components of fitness (a sort of complete compendium of Darwinian selection possibilities), whilst *narrow-sense* natural selection should be taken to comprise things like viability and fecundity selection, as these have been more traditionally considered to be “natural selection,” and the main focus of Darwin’s *The Origin of Species*.

When theorists have considered natural selection acting to oppose sexual selection during the development of a sexual ornament, it is usually in terms of something like viability selection (as an ornament gets bigger, so natural selection via predation acts to limit further exaggeration, for example). If we take Endler’s view, then sexual selection sits within broad-sense evolution by natural selection but is separate from narrow-sense natural selection. However, there is not necessarily a “correct” answer here: we give things names as and when it is useful to do so. We should remember that although partitioning up fitness into different components may be useful, there will be alternative ways to partition fitness. Our definition clarifies a partitioning of fitness that goes back to Darwin. We should also remember that partitioning fitness in theory is more straightforward than doing so in practice. We should not be surprised when the empirical utility of partitioning out fitness is compromised, as when fitness components align and become indistinguishable. This would happen if access to mates correlated perfectly with feeding ability or longevity, for example. Indeed, we should expect such alignments to occur in real mating systems in real ecologies. However, we should not take those failures too seriously either, as concepts such as sexual selection and viability selection still have useful conceptual work to do (for further discussion, see Shuker 2010).

## ALTERNATIVE DEFINITIONS

Here we consider a number of alternative definitions of sexual selection. In Table 2 we provide a flavor of various definitions by a range of authors, from Darwin onwards (for a similar sample, see Alonzo and Servedio 2019). Some of those definitions overlap or restate our definition, and we do not consider those further. We appreciate that our survey is by no means comprehensive, but we nonetheless hope that we cover the major groups of alternative definitions, minor differences in wording notwithstanding.

**Table 2:** Examples of Definitions of Sexual Selection from Darwin Onwards

Darwin 1859, p. 88: “Sexual selection […] depends, not on a struggle for existence, but on a struggle between the males for possession of the females; the result is not death to the unsuccessful competitor, but few or no offspring.”
Darwin 1871, p. 256: “We are, however, here concerned only with that kind of selection, which I have called sexual selection. This depends on the advantage which certain individuals have over other individuals of the same sex and species, in exclusive relation to reproduction.”
Huxley 1938, p. 416: “Darwin’s theory of sexual selection was of the compound deductive-inductive type. Deductively he postulated: (1) that under certain circumstances there would occur a struggle between males for mates, and that the characters giving success in such a struggle would have sexually-selective value and would be perpetuated irrespective of their natural-selective value in the general struggle for existence; (2) that these characters would be of two main types, (a) those subserving male display, (b) those subserving combat between rival males, and that such characters could not be evolved except under the operation of sexual selection as defined by him. With regard to display characters, he further deduced a rudimentary esthetic sense in females, and also a process of female choice as between rival males.”
Ehrman 1972 (Chapter 6 in Campbell), p. 106: “At present it seems best to simply define sexual selection as *all mechanisms which cause deviations from panmixia*.”
Crook 1972 (Chapter 9 in Campbell), p. 264: *Social selection* “is primarily in relation to direct competition.”… “Social selection results from (*a*) effects of competition between the subject and others of either sex with respect of commodities essential to survival in a situation that will allow an attempt at reproduction, (*b*) competition for access to preferred members of the opposite sex for mating and (*c*) effects of competition between subjects for access to commodities in the environment essential for the rearing of their young to reproductive age. Of these *b* is the process most commonly referred to as sexual selection.” [In other words, sexual selection becomes a subset under social selection, but because social selection only relates to direct competition, sexual selection due to scramble or endurance competition is not included].
Maynard Smith 1978 (Chapter 10 Sexual selection, in The Evolution of Sex), p. 168: “As soon as aniosogamy has evolved, different selective forces may act on males and females; it is these differential forces with which I am concerned in this chapter.” [This can thus be read as if Maynard Smith defines sexual selection as selection acting differently on the two sexes].
West-Eberhard 1979, p222 and subsequently: West-Eberhard follows Darwin in viewing sexual selection as competition for mates, but also considers sexual selection a subset of social selection, with the latter characterized by competition within a social group for one or more resources (which might include mates). For example: “I agree with Mayr (1972, p.88) that “something rather important was lost” in the process of redefining fitness and erasing Darwin’s distinction between these two kinds of selection [natural and sexual selection] — just as something is lost by stretching the concept of sexual selection to make it suit new purposes which, however interesting in their own right, tend to obscure what Darwin was trying to say (for example, Ehrman’s1972, p.106, redefinition of sexual selection as “all mechanisms which cause deviations from panmixia,” or Maynard Smith’s, 1978, inclusion of all selection acting differently on the two sexes). When Darwin wrote about sexual selection he focused primarily on *social competition* for mates.”
Partridge and Halliday 1984 (Chapter 9 in Krebs & Davies, 2^nd^ edition), p. 222: “It has long been obvious that the gametes produced in natural populations do not pair up at random. Leaving aside the obvious restrictions imposed by species and gender, some individuals may obtain more fertilizations than others, and particular types of parings may be more common than others. Such nonrandom mating is of fundamental evolutionary importance because different matings may have different fitness consequences.” Continued on p. 225: “As Darwin was first to recognize, variance in the number of successful matings is the raw material for sexual selection, defined as selection on characters giving certain individuals an advantage over others of the same sex in obtaining successful matings.”
Andersson 1994, p. 3: “According to Darwin (1871), sexual selection arises from differences in reproductive success caused by competition over mates.” Continued on p. 8: “Sexual selection of a trait can therefore be viewed as a shorthand phrase for differences in reproductive success, caused by competition over mates, and related to the expression of the traits”; and p. 9: “In spite of many suggestions to the contrary by leading biologists […] the term sexual selection in here restricted to competition over mates.”
Roughgarden et al. 2006, p. 965: “Since 1871, sexual selection theory has often been restated (4), yet contemporary definitions share Darwin’s central narrative: “We now understand… Males, who can produce many offspring with only minimal investment, spread their genes most effectively by mating promiscuously. Female reproductive output is far more constrained by the metabolic costs of producing eggs or offspring, and thus a female’s interests are served more by mate quality than by mate quantity” (5). … The reproductive social behavior of most species has not been studied, but a great many of those that have been do not conform to Darwinian sexual-selection templates. We suggest that sexual selection is always mistaken, even where gender roles superficially match the Darwinian templates.”
Kokko et al. 2006, p. 44: “Sexual selection: selection generated by differential access to opposite-sex gametes (or mates).” [This definition is by far the closest to our definition].
Ritchie 2007, p. 80: “Sexual selection: the component of natural selection arising owing to variation in mating or fertilization success”
Carranza 2009, p. 750: “In 1994, […] I proposed a definition for sexual selection as (page 380; translated from Spanish): ‘those natural selection forces that operate differently in males and females because of the strategies of the sexes’. This is simply to adopt the concept of sex-dependent selection as a modern use of the term sexual selection to investigate the evolution of differences between the sexes.”
Clutton-Brock 2009, p. 8: Contrasts in the operation of sexual selection in the two sexes raise the question of whether adaptations to intrasexual competition in females should be regarded as products of sexual selection or natural selection. In *The Descent of Man* Darwin sometimes described ‘sexual’ selection as selection operating through intrasexual competition to reproduce […] and sometimes as selection operating through competition for mates, although the term is now most commonly restricted to selection operating through intrasexual competition for mating opportunities (Andersson 1994). Because females more commonly need to compete for breeding opportunities than mating opportunities, defining sexual selection in terms of competition for mates has the effect of restricting its operation to males, creating unfortunate dichotomies where functionally similar traits are attributed to sexual selection if they occur in males but to natural selection if they occur in females. […] The most satisfactory solution might be to abandon the distinction between sexual and natural selection altogether and emphasize, instead, the contrasting ways in which selection operates in males and females (Clutton-Brock 2007). However, the distinction between sexual and natural selection is so heavily entrenched that this is unlikely to occur and the most feasible alternative is probably to broaden the concept of sexual selection to include all selection processes operating through intrasexual competition for breeding opportunities in either sex (Clutton-Brock 2007).”
Jones and Ratterman 2009: “Darwin makes it clear that not all selection related to reproduction constitutes sexual selection, as primary sexual traits—like ovaries and testes—can evolve as a consequence of natural selection. Even though he never spells it out in so many words, Darwin’s working definition of sexual selection is essentially identical to the one used by Andersson [1994] and most other scientists studying sexual selection. In particular, ‘‘sexual selection arises from differences in reproductive success caused by competition for access to mates’’ [Andersson 1994, p 3]. This definition admittedly focuses primarily on precopulatory sexual selection, so a more complete definition should also include postcopulatory processes, which can be accomplished by tagging the phrase ‘‘or fertilization opportunities’’ onto the end of Andersson’s definition.”
Kuijper et al. 2012: “Sexual selection is the process by which individuals compete for access to mates and fertilization opportunities.”
Safran et al. 2013, p. 644: …”we define sexual selection as the result of the differential reproductive success that arises from competition for mates and access to fertilizations.”
Rosenthal 2017, p. 503: “Sexual selection. A special case of natural selection: differential reproductive success due to the ability to secure matings and/or fertilization.”
Alonzo and Servedio 2019, Table 1: Their table offers a similar sample of definitions of sexual selection, which (together with our examples above) highlights the challenge for the field of sexual selection.

First, a definition of sexual selection has been proposed that limits itself to mate choice by females or otherwise (Roughgarden et al. 2006). However, as pointed out above, such a definition of sexual selection is far too narrow, as it excludes intrasexual selection via both mating and sperm and egg competition. In particular, it neglects a very substantial body of work that has traditionally sat within the compass of sexual selection: that is intrasexual (such as male–male) contest competition for access to gametes (Andersson 1994; Hardy and Briffa 2013). This component of sexual selection influences traits that are unlikely to have been favored by narrow-sense natural selection, such as horns and antlers (Andersson 1994; Emlen 2008, 2014; see in particular the discussion in McCullough et al. 2016). Focusing on mate choice, although ignoring the intrasexual competition, also lacks conceptual coherence, as it focuses on one group of mechanisms that might influence or mediate within-sex mating competition, whilst not including others (a point made, albeit rather cryptically, by Fisher 1930, p. 131–132).

Second, sexual selection is sometimes defined in terms of variation in reproductive success (for example in textbooks such as Avassar et al. 2013; Clark et al. 2018). Whilst this is understandable in some ways, because mating and reproduction seem to go hand in hand, reproductive success is actually a much broader concept, representing an organism’s direct fitness (in an inclusive fitness framework) through all direct fitness components, including those related to longevity, fecundity, and parental care. Informally, one has to survive to reproduce, as well as find mates, so reproductive success per se is too broad a concept to separate out sexual selection from other aspects of narrow-sense natural selection; all direct fitness collapses into reproductive success. Unfortunately, perhaps the most-repeated of Darwin’s definitions of sexual selection carries with it the sense of “reproductive success,” stating as it does that sexual selection *“depends on the advantage which certain individuals have over other individuals of the same sex and species, in exclusive relation to reproduction”* (Darwin 1871, p. 256; Table 2).

Taken at face value, this definition seems to focus on reproduction, rather than competition for mates (and then gametes). However, Darwin, in the preceding pages, discussed the difference between primary and secondary characters, noting that primary characters – which are needed to reproduce at all – are not the target of sexual selection, but secondary sexual characters are. Darwin also noted that it is “*scarcely possible to decide*” how to identify primary versus secondary sexual characters or separate out the effects of natural or sexual selection. So, the difficulties in ascribing forms of selection to traits were appreciated from the very outset of the intellectual history of sexual selection (Darwin 1871, p. 254; see also Fisher 1930, p. 132).

The relationship between reproductive success and sexual selection has been brought into renewed focus in recent years through a number of papers that have sought to redraw, or at least reexamine, ways in which sexual selection may influence females (Clutton-Brock 2007, 2009, 2010, 2017; Rosvall 2011; Stockley and Bro-Jørgensen 2011; Tobias et al. 2012). From a series of observations of – mostly – vertebrates, the idea that individuals of a given sex compete between themselves for resources, or social status, crucial for reproduction has been suggested to be a form of sexual selection, with the idea that “reproductive competition” comes to replace or broaden the notion of “competition for mates.” The problem with bringing female-female competition for reproductive resources, or indeed male–male competition for such resources, into sexual selection is that much of what all organisms do – male or female – is to compete for resources that sooner or later contribute to variation in reproductive success. In the broadest terms, this means that natural and sexual selection perfectly coincide, and the latter term becomes meaningless (Clutton-Brock 2007, 2009; Shuker 2010). If we wished to be less broad, then we would have to decide, a priori, how “close” to reproduction the resource was, or the competition for it was, when saying whether or not that competition engenders natural or sexual selection. Given that resource acquisition, and the life-history decisions that underlie how resources are allocated and competed for, may play out over the long-term (for example early-life effects: Lindström 1999; Jonsson and Jonsson 2011), teasing resource competition apart to assign to sexual or natural selection would be challenging. Instead, we suggest that components of “natural selection” are about being able to enter and remain in the *fertilization game*, where competition for access to mates and their gametes occurs, and that “sexual selection” is about how well you succeed in that game when compared with other same-sex contestants. Whilst fully recognizing the impact sexual selection has on the fitness of both sexes, we consider that *competition for access to gametes* is the focus of sexual selection, and that competition for resources – whether directly required for reproduction or not – is the focus of other components of fitness, *unless* the resource itself influences access to gametes. We will return to this topic below.

Another definition of sexual selection is again drawn from a particular reading of Darwin. Authors such as Padian and Horner have argued that sexual selection is characterized by sexual dimorphism of secondary sexual traits (Padian and Horner 2011a, 2011b, 2014a, 2014b; but see Knell et al. 2013; Borkovic and Russell 2014; Clutton-Brock 2017 also discusses the links between sexual dimorphism and sexual selection). Basing this idea on the detailed discussions of dimorphism given by Darwin, sexual selection is then defined either as the driver of sexual dimorphism, or only as occurring when there is sexual dimorphism (for example Padian and Horner 2014b). Put another way, without sexual dimorphism, sexual selection cannot act (or be said to have acted). This view of sexual selection, which we reject, has been discussed in particular in the paleontological literature, where evidence for sexual selection is notoriously difficult to find (for example Knell et al. 2013; Mallon 2017; Hone and Mallon 2017; O’Brien et al. 2018). However, that search is made harder still by stipulating a priori that fossils need to exhibit sexual dimorphism before sexual selection as a mechanism can come into play. Whilst there is much that could be discussed here, we will simply make the following points. First, there is clear evidence that sexual dimorphism can arise through narrow-sense natural selection on the sexes favoring ecological displacement (Shine 1989; Fairbairn et al. 2007). Second, the requirement for sexual dimorphism would mean that sexual selection cannot occur in isogamous species with separate mating types, or indeed in simultaneous hermaphrodites. Third, there is clear evidence of sexual selection in species that are sexually monomorphic, such as mutual mate choice for head ornaments in crested auklets (Jones and Hunter 1993). All in all, the requirement for sexual dimorphism for sexual selection to be said to be occurring is overly restrictive and lacks logical coherence when considering the competition for gametes in monomorphic species.

Along similar lines, but not so closely tied to sexual dimorphism per se, another alternative definition focuses on the different patterns of selection that arise on the two sexes (Carranza 2009), taking its cue from the title of Darwin’s book (1871: “*selection in relation to sex”*). As noted above, we should be careful in reading too much into the title of that book, and indeed the phraseology Darwin used at times, given the social circumstances under which he was writing, and the pressures applied by his publisher (Dawson 2007). Nonetheless, it is still not clear that viewing sexual selection in terms of differential patterns of selection on males and females is useful. Carranza (2009) argued for a definition of sexual selection based on the differences between the sexes in the action of selection. We think that this definition is a long way from Darwin’s conception of sexual selection and sits more neatly alongside the current body of sexual conflict theory (Parker 1979; Arnqvist and Rowe 2005). As a definition of sexual selection – at least as usually envisaged – it is problematic as the sexes may experience different patterns of selection on traits unrelated to reproduction (that is ecological causes of sexual dimorphism, as discussed above: Shine 1989). The corollary of this, as Carranza himself suggests, is that once we have separate sexual functions, then nearly all selection may be sexual selection. As such, sexual selection swallows natural selection (much as we saw natural selection swallowing sexual selection above). Therefore, whilst sexual selection may contribute to the opposing patterns of selection that arise on males and females (that is, sexual conflict), sexual conflict is most decidedly *not* the same thing as sexual selection (Shuker 2010; Kokko et al. 2014).

The final alternative definition of sexual selection we wish to consider explicitly is of a somewhat different nature: defining sexual selection in terms of one way in which it may be measured (Shuster and Wade 2003; see suggestion in Roughgarden et al. 2015). Shuster and Wade (2003; henceforth S&W) summarized a research programme initiated by Wade and coworkers that sought to quantify the differences between males and females in the variance in the number of mates each sex obtained (Wade 1979; Wade and Arnold 1980; Wade 1995). More formally, after Crow (1958, 1962), they show that the difference between males and females in the total opportunity of selection (*I*_males_ - *I*_females_) is equal to what is termed *I*_mates_, where *I* represents the opportunity for selection. S&W state that *I*_mates_ gives a *“standardized measure of the intensity of sexual selection on males and the sex difference in strength of selection”* (Shuster and Wade 2003, p. 29). Importantly, as the authors also note, the opportunity for selection is just that, only the opportunity. As such, *I*_mates_ offers only an upper limit on selection.

The *I*_mates_ approach has been the subject of a number of critiques and rebuttals down the years (for example Sutherland 1985; Downhower et al. 1987; Shuster and Wade 2003; Klug et al. 2010; Krakauer et al. 2011; Jennions et al. 2012; Henshaw et al. 2016). We do not wish to rehearse that debate here. Rather we wish to argue against the use of this measure as a *definition* of sexual selection. First, it is true that one cannot measure what one cannot define. But to define something by its measurement is a different thing entirely, risking circularity and reification. Second, and more problematic, is that the opportunity for sexual selection may also include random processes that influence the variance in obtaining gametes within a sex, so it may be nonzero even in the absence of any form of selection.

## THE ROLE OF FEMALES IN SEXUAL SELECTION

Before we challenge our definition in the face of some real-world complications, we need to address the recent literature that has attempted to redraw the scope of sexual selection that we have outlined above, in particular in terms of accommodating female “reproductive competition” within a framework of sexual selection. Clearly females can and do compete for male gametes, but as noted above females also compete for other resources required for reproduction, and it is “reproductive competition” in this context that authors such as Clutton-Brock (see above) have suggested should be included with sexual selection. The argument is that by neglecting this kind of female–female competition, we are missing something important about sexual selection. We disagree and here we extend our argument introduced above.

**Figure 1:**
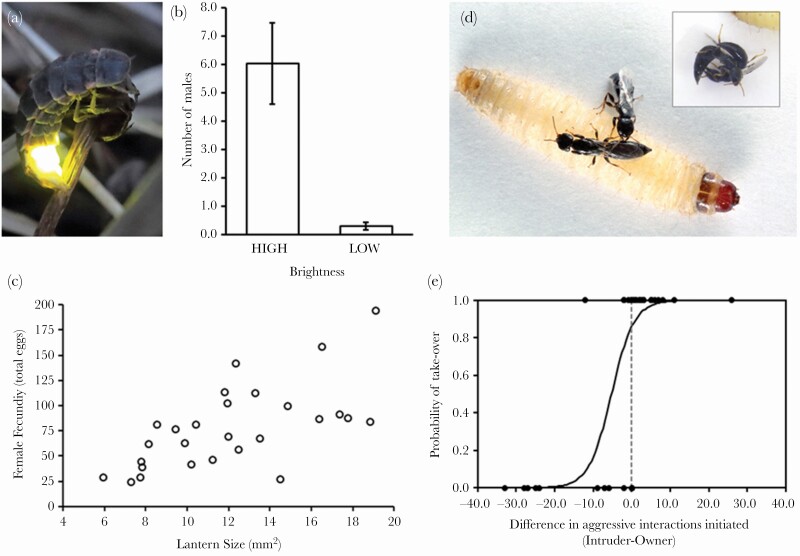
Female competition for (a–c) gametes in the common glow worm, and (d–e) resources required for reproduction in the parasitoid wasp *Goniozus legneri*. (a) A female *Lampyris noctiluca* glowing to attract mates (photo: Jouni Valkeeniemi). (b) Experimental evidence that male *L*. *noctiluca* are attracted to brighter green LED lights, used here to mimic female displays (High brightness and Low brightness are 12.6 × 10^12^ and 7.0 × 10^11^ photons cm^−2^ s^−1^ respectively), whilst females with larger lanterns are also more fecund (c). (d) Female *G*. *legneri* fight for possession of a host on which to lay eggs, which can lead to vigorous struggles (inset; photos: Sonia Dourlot). (e) These contests are determined by differences in aggression between the owner and intruder, with the more aggressive individual, that initiates more interactions, winning the resource. The fitted line is from a logistic regression. (b-c) Figures redrawn from Open Access Data provided by Hopkins et al. (2015) under a CC0 1.0 Universal Public Domain Dedication license. (d–e) Figures reproduced from Goubault et al. (2006) with permission of The Royal Society.

First, our definition is explicitly sex and sex-role neutral: there is nothing in our definition that precludes females, or indeed hermaphrodites, or isogamous species, from the action of sexual selection. A recent example of females competing for access to male gametes comes from the common glowworm, *Lampyris noctiluca* (Hopkins et al. 2015; Borshagovski et al. 2019; Figure 1a–c). In this species, the female attracts a male by glowing. There is no resource other than sperm that successful females gain, and far from all females manage to attract a male. Thus, clearly female ability to attract males by their glow is a sexually selected trait.

Second, we do not consider that there is anything particularly special, intrinsically better, or more interesting, about sexual selection compared with other forms of selection. As such, we see no detriment – theoretically or empirically – in whether certain forms of competition are or are not included within the scope of sexual selection. What is more important is a coherent and robust definition of sexual selection and the resulting body of work. For instance, that female *Goniozus* wasps fight over access to hosts, and that this competition determines their ability to reproduce (Goubault et al. 2006, 2007; Figure 1d–e) resulting in narrow-sense natural selection, is just as fascinating as male red deer competing over access to a harem of females (Clutton-Brock et al. 1982) resulting in sexual selection. All organisms compete for the *opportunity* to reproduce, throughout their development, competing for the necessary food and other resources prior to reproductive maturity, fighting off parasites and predators, and so on. For female *Goniozus* wasps (Figure 1d), the final arena of reproductive competition shares much in common with contest competition over access to gametes, but what is being competed over is different: it is not gametes, but rather the resources needed for offspring to develop, in their case lepidopteran larvae. In this regard, the resulting selection is akin to selection on mammals to supply young with food and protection, the selection that we usually ascribe to narrow-sense natural selection. Apparent similarities (for example, in terms of contests) should not get in the way of the underlying logic of what is being competed for and why. As a hypothetical test (similar to the logic of potential reproductive rates: Clutton-Brock and Parker 1992), we may consider what would happen if an individual were given access to an extra set of gametes, what would happen to their fitness? In the red deer case, it would clearly increase male fitness, whereas in the female wasp case it would not, as the latter are limited by hosts, not gametes.

Another example is reproductive dominance. Reproductive dominance can achieve two aims. First, it can prevent other same-sex individuals from producing gametes at all. Well-known examples are found in animals that live in close-knit communities. For example, in ants and meerkats, dominant females may prevent other females in the colony from developing eggs or evict subdominants (for example Bourke and Franks 1995; Young et al. 2006; Clutton-Brock et al. 2008). In these instances, reproductive dominance is primarily about policing the *opportunity to reproduce*, limiting competition for the resources that offspring need, and so maximize relative fecundity of the dominant females. It is not about access to opposite-sex gametes, as subordinates have been precluded from even entering into that competition. Our conclusion, therefore, is that it falls under narrow-sense natural selection, and reproductive competition is a useful label to describe it (for example Clutton-Brock 2017).

Second, reproductive dominance can prevent other *reproductively competent* individuals in the group from mating and competing for fertilisation. In chimpanzees, all mature males are sexually competent, and indeed may attempt to copulate with a female on “consortships” away from the group, but the dominant alpha male will otherwise police the sexual behavior of subordinate males. Here, the reproductive dominance is sexually selected, as the dominant male is preventing subordinates from accessing opposite-sex gametes, subordinates who are otherwise able to inseminate females. Therefore, to repeat the hypothetical experiment, if a subdominant meerkat female is given (secretly) free access to male gametes, then it presumably will not improve her fitness, as she does not have sufficient status in the group to reproduce, regardless of access to gametes, whereas a free mating for a chimpanzee male is free fitness.

Similarly, in harem-holding sequential hermaphrodites, such as many wrasses, the largest and most dominant individual in a shoal becomes a male, whereas all the subdominant individuals reproduce as females. Here, all individuals are able to reproduce, but by being dominant, the male function gets exclusive access to the gametes of the other sex (hence sexual selection). Here the competition for access to gametes becomes tied up with which is the optimal sex to be if dominant.

We do not pretend that the dividing line will necessarily be easy to draw in real organisms in all cases. In the pipefish *Syngnathus typhle*, males provide care by carrying the embryos in a brood pouch on the tail. In this species, the hypothetical experiment above has already been carried out, and it shows that females benefit more from free access to the opposite sex and its gametes than males do, and that, on average, female egg production is almost twice as high as the male capacity to care for the eggs (Berglund et al. 1989), creating female-female competition for mating opportunities with males (Rosenqvist and Berglund 2011). Fish typically grow throughout their life and this species is no exception. Experimental work shows that small (young) females with free access to males reduce egg production and instead invest more in growth in the presence of an enclosed but visible large female, compared with when kept in the visual presence of a small female, or in the absence of other females (Berglund 1991). Arguably, this is a result of intrasexual dominance by the large female, combined with a male preference for larger females that produce more and larger eggs, favoring a life-history decision by the small female to allocate her resources to increasing her odds at gaining mating success later in life. We view this as an example of female-female competition for mates, in which success is achieved, not through combat, but through reproductive dominance and competition in attractiveness. Nevertheless, because males provide both gametes and care, females compete for both gametes to fertilize her eggs (sexual selection) and for male care (natural selection; male care increases the growth and survival of the female’s offspring: Nygård et al. 2019).

**Figure 2:**
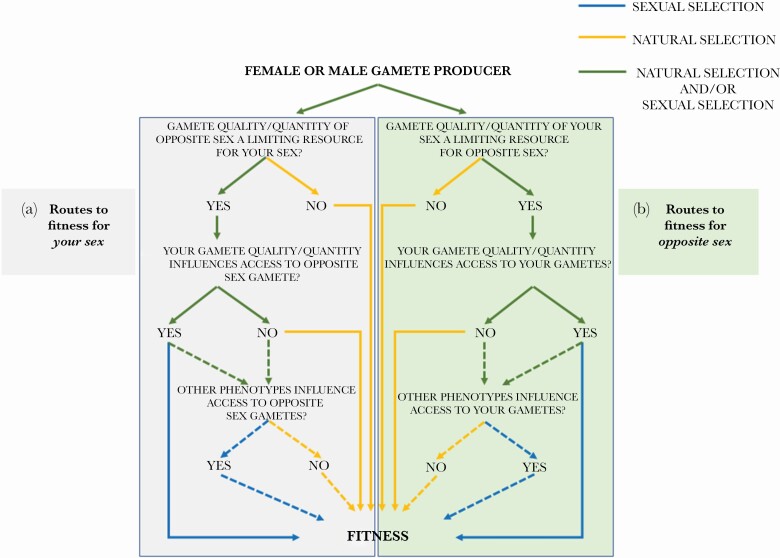
Routes to fitness for female and male gamete producers. (a) Routes to fitness for your sex, and (b) routes to fitness for the opposite sex. The two sexes are completely interchangeable. In (a), individuals and their gametes of the focal sex are the subject of competition, and in (b) they are the object of competition. As such, in (a) selection arises within the focal sex, and in (b) selection arises within the opposite sex, as a result of gametic traits and other phenotypes in the focal sex. Both (a) and (b) will occur concurrently. Yellow lines denote routes to fitness via narrow-sense natural selection, blue lines denote routes to fitness via sexual selection, and green lines denotes routes where both natural and sexual selection may yet occur. Solid lines denote fitness routes influenced by gamete quality or quantity in the focal sex, and dotted lines denote fitness routes influenced by other traits.

We reiterate that we are in no way seeking to exclude females from sexual selection, nor to give males special status in terms of the action of sexual selection. Indeed, after Eberhard (1996), we agree that females may still remain under-appreciated in terms of how females influence sexual selection on males, and in turn how sexual selection acts on females. For instance, the awareness that male mate choice (leading to sexual selection on *females*) is much more common than we used to think attests to this (Bonduriansky 2001; Edward and Chapman 2011). However, that does not mean that female-female reproductive competition should necessarily be considered sexual selection by default. For us, what matters is what is being competed over. It is competition for fertilizations that yields sexual selection, and by making this distinction we can recognize shared or different *routes to fitness* for females and males (Figure 2).

## CHALLENGING OUR DEFINITION

We wish to finish with four challenges to our definition of sexual selection that we have not already explicitly considered. To do this, we will outline some other hypothetical empirical scenarios and identify what is and is not sexual selection under our definition, highlighting areas of tension in interpretation that may arise. We appreciate that there are other scenarios, such as infanticide, that we do not treat here. For each of these scenarios, we assume relevant genetic variation underlying the traits of interest, so for our purposes here selection equates with evolution by natural and/or sexual selection. Throughout, it is key to identify what is being competed for, and by whom. Figure 2 provides a guide to help identify different “routes to fitness.” Crucially, a gamete producer can be both the target of competition for the other sex, and also a competitor for the gametes of the other sex.

### Endurance rivalry

Endurance rivalry describes competition for gametes when the amount of time invested in gaining fertilizations is how fitness is obtained (Thornhill and Alcock 1983; Andersson 1994). Endurance rivalry may play out over hours (for example in short-lived swarming insects), or over longer time periods (such as lek attendance during a breeding season in species such as ruff), including repeated breeding events across a lifetime. Given that it is often hard to follow individuals across their whole lifetime in the field, endurance rivalry – basically, living and mating for as long as you can – may be a rather common sexual selection fitness component. In such cases, longevity and mating success will be positively correlated, and the roles of natural and sexual selection will be hard to separate. Put another way, longevity will be both sexually and naturally selected.

We could of course consider that underlying both mating success and longevity are a series of allocation trade-offs, such that an organism that did not have to compete for mates would live longer (although we may also see positive correlations between these traits among individuals: van Noordwijk and de Jong 1986). If access to mates or resources were manipulated experimentally, then we could estimate the proportion of fitness due to natural or sexual selection fitness components, but it would not always be straightforward. As discussed above though, we do not consider this a grave problem. We should in fact expect any definition of sexual selection to allow other fitness components to align and confound it. Traits will be under many possible forms of natural and sexual selection, as many aspects of an organism’s biology and ecology influence fitness (for example Evans and Garcia-Gonzalez 2016).

### Mate choice: the chosen sex

Mate choice means, by definition, that there is nonrandom fertilization success with respect to one or more phenotypes amongst members of the *chosen* sex, and so there is sexual selection acting on that sex. All members of the *choosy* sex may prefer the same few individuals, or indeed the same single individual, which will dictate how strong sexual selection on the chosen sex is. Alternatively, the choosy sex may differ amongst themselves in their preferences. This could of course lead to complex patterns of sexually selected fitness amongst the chosen sex. Nonetheless, this situation still seems relatively straightforward.

However, what happens if we focus on what is being chosen and what the routes to fitness are (Figure 2)? Under our definition the sexes are interchangeable, but for simplicity, consider choosy females and chosen males. First, consider that males vary in terms of a resource that they have – this might be a territory or an investment made in parental care. As before, this is quite simple, with males being chosen if they hold a territory or provide parental care, and so territorial or parental care investment is under sexual selection in *males*. Males may also vary in terms of the quality of their resource or their care, and again females may prefer to fertilize their eggs with males with higher quality resources or higher quality parental care. This would lead to males evolving to provide more care or hold larger territories than expected based only on naturally selected fitness components (Kvarnemo 2006), potentially affecting male survival, offspring survival, as well male mating success. The overall result is the same though: sexual selection on males, with some doing better at getting fertilizations than others due to mate choice. This means that resources, and competition for those resources, can influence and be part of sexual selection on males. However, it is only the extent to which providing those resources influences a male’s *access to gametes* that is sexually selected.

Following on from this, if choosy *females* start competing amongst themselves for the male that provides the most care or holds the best territory, these females are under natural selection, as long as the target of the competition is access to the *resource*, and not to *gametes*. This means that the bushcricket *Kawanaphila nartee*, a classic example of sexual selection on females, generated by female-female competition for mating opportunities early in the mating season when food availability (pollen) is limited (Gwynne and Simmons 1990; Simmons and Bailey 1990), may need to be reevaluated: If females compete for males to gain access to their gametes, they are then under sexual selection, but as soon as females instead compete for males to gain access to their nutritious spermatophylax, and not their gametes, this competition falls under natural selection in our proposed framework. That said, male ability to locate pollen needed to produce the spermatophylax, without which a male bushcricket cannot mate, would be a sexually selected trait in males when it leads to greater access to female gametes. A tangled bank indeed!

Crucially, what this means is that resources can have *both* primary and secondary sexual function, as with genitalia. An animal needs resources to survive and produce offspring in the first place, as they also need genitalia, but this is natural selection on primary sexual function, the selection that means an animal is able to *engage in* competition for gametes. If resources or genitalia then differentially influence access to gametes, they gain secondary sexual function and come under sexual selection. And there is now abundant evidence that sexual selection has driven divergence in animal genitalia, including in terms of rapid evolutionary change, in both males and females (for example Eberhard 1996; Simmons 2014; Simmons and Fitzpatrick 2019; Sloan and Simmons 2019).

Next, we will consider the choosy sex, and how choice itself arises.

### Mate choice: the choosy sex

Typically, the selection that causes a sex to become choosy in the first place is natural selection, i.e. there is a direct, natural selection fitness benefit to discriminating among potential partners (Fisher 1930; Andersson 1994). However, how does sexual selection play out amongst the choosy sex once that sex has become choosy? Again, we will consider females as the choosy sex, but as before, the sexes are interchangeable. Under our definition, sexual selection acts on the choosy sex if the behavioral, morphological or physiological traits that generate choice mean that females differ nonrandomly in *their* access to gametes. Mate choice is not always viewed in this competitive way among the choosers, and indeed it may not be competitive. For instance, one male may be super-attractive and also be able to inseminate fully all females in the population, and so all the females mate with him, their preferred male. Whilst there would be extremely strong sexual selection amongst males, given the extremely high reproductive skew, there would be no sexual selection amongst females, as there are *no limiting* resources in terms of *gametes* to compete over.

On the other hand, male gametes may be limiting in terms of their quantity or quality, and so competition amongst the choosy sex will arise. That competition can manifest itself as a form of – in this instance – female-female contest or scramble competition (depending on the mating system), or be influenced by patterns of male mate choice. Although rarely thought of in this way, female ability to store sperm may also be sexually selected if good storage allows the female prolonged access to a preferred male’s gametes.

We can imagine a variety of outcomes, all of which will be contingent on aspects of the mating system, which we will treat here with a broad brush. Females that have very wide acceptance thresholds may be more likely to end up with a lower quality male than a stricter chooser, and so the latter will be favored by sexual selection if they nonrandomly gain access to higher quality gametes. On the other hand, a stricter chooser may also miss-out on mating entirely, in which case the more permissive females will be favored. Thus, whether choosiness is favored or disfavored by sexual selection in the choosy sex will depend on how and why the chosen sex is a limiting resource. Both mate availability and mate quality may act in concert in shaping the level and extent of choosiness (Parker 1978, 1983; Owens and Thompson 1994; Parker and Partridge 1998). Mating failure – the failure to mate and/or be inseminated – will therefore be selected against by sexual selection, as the focal individual (of whichever sex) has failed to gain sufficient access to gametes to produce offspring (Garcia-Gonzalez 2004; Rhainds 2010; Greenway et al. 2015, 2017).

So far, we have considered choice in fairly simple terms, implicitly suggesting some sort of “granting of access” to allow a mating or transfer of gametes to take place, or perhaps choosing one stored ejaculate over another, and using that to fertilize eggs. However, what if choice is mediated by a change in resource allocation, such that the choosy sex invests more in the reproductive event because of the identity of the chosen partner (differential allocation: Burley 1986, 1988; Sheldon 2000)?

The least problematic example would be if a male releases more sperm with an attractive and/or high quality female, investing more in the given mating to protect and promote chances of paternity with a high quality partner. This sort of strategic sperm allocation commonly occurs (Simmons 2001; Wedell et al. 2002; Parker and Pizzari 2010), and it could generate sexual selection in females as well (see above). The extent to which such male investment increases access to female gametes is the extent to which that investment is sexually selected in males. However, if a female exerts choice by releasing more eggs (or by fertilizing more eggs than she would with another male), how do we interpret this? Under our view, by making more gametes available to the chosen male, whatever phenotype in the *male* is non-randomly associated with this choice by the female is therefore under sexual selection. In other words, that male phenotype has allowed him greater access to gametes than another male would have gained. It does not matter that the number of eggs that become available was only decided during or after the mating. Unsurprisingly, Eberhard (1996) includes just such a mechanism of choice in his list of possible mechanisms of cryptic female choice.

Differential allocation is perhaps more complicated from the female perspective though, as increasing the number of eggs (that is increasing *fecundity*) in response to a male trait of her liking is usually associated with narrow-sense natural selection, not sexual selection. For females in this case, our definition suggests the following. The extent to which females increasing the numbers of eggs produced with a given male means that they have greater access to male gametes as a limiting resource (either in terms of quantity or quality of males and their sperm) is the extent to which this behavior is sexually selected in *females* (Figure 2). In other words, if females nonrandomly increase their immediate fecundity with respect to male phenotype (that is they exhibit choice), and by doing so they limit the access to preferred males for other females, then that behavior is sexually selected. Remember, sexual selection requires competition, and competition requires a limiting resource; if females increasing their reproductive allocation with a given male does not influence his availability to father offspring of other females, then *her* allocation decisions are *naturally selected* and only about her *fecundity* under our definition. In summary, then, differential allocation can be a mechanism of sexual selection in both sexes, and a way of expressing mate choice, but only if it leads to greater access to gametes of the opposite sex.

### Mate choice: the chooser and the chosen

Finally, imagine a species where both sexes choose mates and provide parental care. The overall reproductive success of the pair depends on how much each individual is prepared to invest in gametes, brood provisioning, and other forms of care (Clutton-Brock 1991; Royle et al. 2012). In species such as this, we would expect both sexes to be both chooser and chosen at the same time, although in truth, mate choice by both sexes, regardless of patterns of parental care, may be more common than is often thought (for example Bonduriansky 2001; Edward and Chapman 2011; Rosenthal 2017). Generally, and ignoring complicating trade-offs, we would expect increased fecundity to be favored by natural selection, so diligent parents that worked well together and raised a large brood would be favored by natural selection to the extent that their investment led to greater direct fitness than that of other pairs. Yet after our rationale, there is the scope here for sexual selection as well, if the way in which resources and parental investment are allocated influences whether or not one or both sexes do better in competition for gametes (Figure 2).

This competition could arise, for instance, if there are repeated breeding attempts and/or the opportunity for extra pair offspring, whereby investment in reproduction nonrandomly influences access to gametes for a given individual. Investment in the breeding attempt may then have three functions. First, it increases reproductive success (and so is under natural selection). Second, it increases access to gametes (as ever, in terms of quality or quantity), either by encouraging the partner to make more gametes available, or by discouraging the partner to make gametes available to others (and so is under sexual selection). Third, it may increase access to gametes by attracting other partners for extra pair fertilization (again under sexual selection). Only under strict monogamy, with no divorce, could we remove the possibility of sexual selection on resource allocation or parental investment during rearing, with only sexual selection before pairing being possible.

There are analogies with the debate over the role of nuptial gifts in species where males provide females with food or nutrients which are consumed by the female, during or after insemination (Gwynne 1990; Wedell 1993; Simmons 1995; Vahed 1998; Lewis and South 2012). In instances such as this, the investment a male makes in his nuptial gift may be considered as either (a) parental investment, as the energy and resources passed to the female help the female provision his future offspring (and so is under narrow-sense natural selection), or (b) sexually selected, as a bigger gift means the female makes more eggs available for his sperm to fertilize and/or allows more of his sperm to enter into sperm competition to fertilize her eggs. Note that under our definition, the extent to which males vary in how their gifts cause females to “make more eggs available” is a source of sexual selection in males, even if in doing so a direct fecundity benefit also accrues to the females. Put another way, the natural selection fitness component we call “fecundity selection” only pertains to females – females have fecundity, males have access to gametes. Yes, males can run out of sperm, but that is fertility, not fecundity. The nuptial gift literature has not always made this distinction clear though, associating the increased access of male sperm to females able to produce more eggs as a result of nuptial gifts with natural selection, rather than sexual selection.

In truth, nuptial gifts may well serve both parental investment and sexual selection functions, as data from the bushcricket *Requena verticalis* suggest for example (Simmons et al. 1993), and only with very careful experimentation and manipulation could one be able to identify the extent to which a nuptial gift brings fitness to males via one route or another. Without such experiments, arguments over the function of nuptial gifts may be unproductive. Worse still, the two functions may also not be fully independent: greater parental investment may also select for greater investment in sperm competition mechanisms to protect that parental investment (Lewis and South 2012).

Finally, in Arnold and Wade’s influential paper (1984, their Figure 1), the process resulting in large males mating with a large number of females is labelled sexual selection, but when larger males were better able to mate with more fecund females, it is called it natural selection. Under our definition, however, both cases are examples of sexual selection on males.

Importantly, as we have stressed repeatedly above, we do not pretend that it will be empirically simple to disentangle the contribution of natural and sexual selection (and it may not even be interesting to do so), but this is where the logic takes us. We admit that in the process of writing this paper we have had to “sacrifice” some of our own favorite examples of sexual selection. Having to give them up meant that we only reluctantly went where the logic took us.

## Conclusions

We have presented a definition of sexual selection and sought to put that definition into context. It is not a new definition, being strongly grounded in Darwin’s writings, and being the definition promoted by Andersson (1994), Kokko et al. (2006), and emphasized by Shuker (2010; his “consensus definition,” see Roughgarden 2015). We have explored alternative definitions, in particular those associated with a role for female resource competition and with the measurement of sexual selection. We believe the definition presented here has many benefits, including being sex- and sex-role neutral, and agnostic about other components of fitness. By doing so, it also acknowledges (or rather allows) sexual selection to be empirically tangled up with other fitness components that will make the measurement and interpretation of sexual selection problematic. We should expect that though, and in such cases the utility of holding to our names for individual fitness components starts to be called into question anyway; all evolutionary biologists are population geneticists at heart, whether they like it or not. Our main aim in writing this paper though is to allow the next generation of sexual selection researchers to address the many questions still left unanswered without the baggage of the definition of sexual selection left lying around. Sexual selection may be complicated, but its definition, we believe, is simple.
